# Radiomics of Tumor Heterogeneity in Longitudinal Dynamic Contrast-Enhanced Magnetic Resonance Imaging for Predicting Response to Neoadjuvant Chemotherapy in Breast Cancer

**DOI:** 10.3389/fmolb.2021.622219

**Published:** 2021-03-22

**Authors:** Ming Fan, Hang Chen, Chao You, Li Liu, Yajia Gu, Weijun Peng, Xin Gao, Lihua Li

**Affiliations:** ^1^ Institute of Biomedical Engineering and Instrumentation, Hangzhou Dianzi University, Hangzhou, China; ^2^ Department of Radiology, Fudan University Shanghai Cancer Center, Shanghai, China; ^3^ Computational Bioscience Research Center (CBRC), Computer, Electrical and Mathematical Sciences and Engineering Division (CEMSE), King Abdullah University of Science and Technology (KAUST), Thuwal, Saudi Arabia

**Keywords:** dynamic contrast-enhanced magnetic resonance imaging, breast cancer, neoadjuvant chemotherapy, volumetric change, feature change

## Abstract

Breast tumor morphological and vascular characteristics can be changed during neoadjuvant chemotherapy (NACT). The early changes in tumor heterogeneity can be quantitatively modeled by longitudinal dynamic contrast-enhanced magnetic resonance imaging (DCE-MRI), which is useful in predicting responses to NACT in breast cancer. In this retrospective analysis, 114 female patients with unilateral unifocal primary breast cancer who received NACT were included in a development (*n* = 61) dataset and a testing dataset (*n* = 53). DCE-MRI was performed for each patient before and after treatment (two cycles of NACT) to generate baseline and early follow-up images, respectively. Feature-level changes (delta) of the entire tumor were evaluated by calculating the relative net feature change (deltaRAD) between baseline and follow-up images. The voxel-level change inside the tumor was evaluated, which yielded a Jacobian map by registering the follow-up image to the baseline image. Clinical information and the radiomic features were fused to enhance the predictive performance. The area under the curve (AUC) values were assessed to evaluate the prediction performance. Predictive models using radiomics based on pre- and post-treatment images, Jacobian maps and deltaRAD showed AUC values of 0.568, 0.767, 0.630 and 0.726, respectively. When features from these images were fused, the predictive model generated an AUC value of 0.771. After adding the molecular subtype information in the fused model, the performance was increased to an AUC of 0.809 (sensitivity of 0.826 and specificity of 0.800), which is significantly higher than that of the baseline imaging- and Jacobian map-based predictive models (*p* = 0.028 and 0.019, respectively). The level of tumor heterogeneity reduction (evaluated by texture feature) is higher in the NACT responders than in the nonresponders. The results suggested that changes in DCE-MRI features that reflect a reduction in tumor heterogeneity following NACT could provide early prediction of breast tumor response. The prediction was improved when the molecular subtype information was combined into the model.

## Introduction

Neoadjuvant chemotherapy (NACT) is commonly used in treatment of locally advanced or large operable breast cancers with the aim of downstaging before surgery (Taghian et al., 2004; Kaufmann et al., 2007). The achievement of a pathologic complete response (pCR) is associated with improved survival in patients with breast cancer (Cortazar et al., 2014). Despite the benefit, a subset of patients may experience a failure of treatment and suffer from the side effects of NACT. Therefore, accurate determination of the outcome of NACT is of vital importance for tailored treatment of patients with breast cancer.

Dynamic contrast-enhanced magnetic resonance imaging (DCE-MRI), which is routinely used in clinical practice, provides morphological tumor characteristics and functional information about the blood flow, vascular status and angiogenesis (Pinker et al., 2017; Mann et al., 2019). A systematic review demonstrated that MRI-based radiomics achieved overall promising performance in NACT response prediction (Granzier et al., 2019) and residual tumor size evaluation (Kim et al., 2018a), while a DCE-MRI-based predictive model achieved better accuracy than a model using other parametric images (Fowler et al., 2017). Radiomics features derived from the pretreatment MRI have been used for predicting response to NACT for breast cancer (Uematsu et al., 2010; Braman et al., 2017; Santamaría et al., 2017; Reig et al., 2020). Our previous study used DCE-MRI to identify and validate predictive imaging biomarkers for NACT using two datasets with different imaging protocols for training and testing (Fan et al., 2017). These studies were performed using radiomics of preoperative breast MRI without considering the imaging features of longitudinal changes in MRI features that could be promising in predicting tumor responses to NACT.

The NACT regimen usually takes six to eight cycles to finish the whole treatment procedure. Longitudinal imaging is usually performed during the procedure to monitor and evaluate treatment response. The changes of tumor heterogeneity in DCE-MRI between the preoperative and early NACT (e.g., two cycles of treatment) may provide information for early prediction of the eventual treatment outcome. Previous studies have demonstrated evidence of longitudinal changes in pharmacokinetic parameters (Dogan et al., 2019), tumor sizes (Tudorica et al., 2016), and tumor MRI texture parameters (Parikh et al., 2014; Henderson et al., 2017; Eun et al., 2020; Nadrljanski and Milosevic, 2020) being correlated with responses to NACT in breast cancer patients. These studies mainly analyzed the feature-level changes of the heterogeneity by evaluating longitudinal images within a tumor. Despite the advances of these methods, the voxelwise changes inside a tumor between baseline and post-NACT MRI scans may not be captured by feature analysis of the entire tumor.

To this end, attempts have been conducted by aligning intermediate MRI to baseline images to evaluate changes in tumor heterogeneity in a voxel-by-voxel manner. A previous study implemented an accurate image registration technique using a parametric response map (PRM), which can provide quantitative voxel-based information regarding heterogeneous changes within the tumor during treatment (Galban et al., 2011; Galban et al., 2012; Cho et al., 2014). The nonrigid nature of the human breast requires methods using deformable registration of longitudinal tumor changes during NACT (Li et al., 2009; Ou et al., 2015). A recent study uses deformable methods to capture tumor heterogeneity for early prediction of response to NACT (Jahani et al., 2019). However, whether the quantitative evaluation of longitudinal tumor changes by radiomic analysis is associated with tumor responses is still unclear.

To predict NACT responses in breast cancer, changes in tumor heterogeneity were evaluated both in voxel-by-voxel and feature-level manners using longitudinal DCE-MR images. Radiomic features were extracted at baseline and post-treatment images and the voxel-level map of volumetric change before and after early NACT. Additionally, feature-level changes in tumor heterogeneity were evaluated by calculating the relative net radiomic feature change between baseline and follow-up images (deltaRAD). Predictive models were then established using the radiomic features derived from these images. Our comprehensive analyses demonstrated how the heterogeneity changes in DCE-MRI before and after early NACT could affect the accuracy of prediction of the response to NACT.

## Materials and Methods

### Framework Overview

**FIGURE 1:**
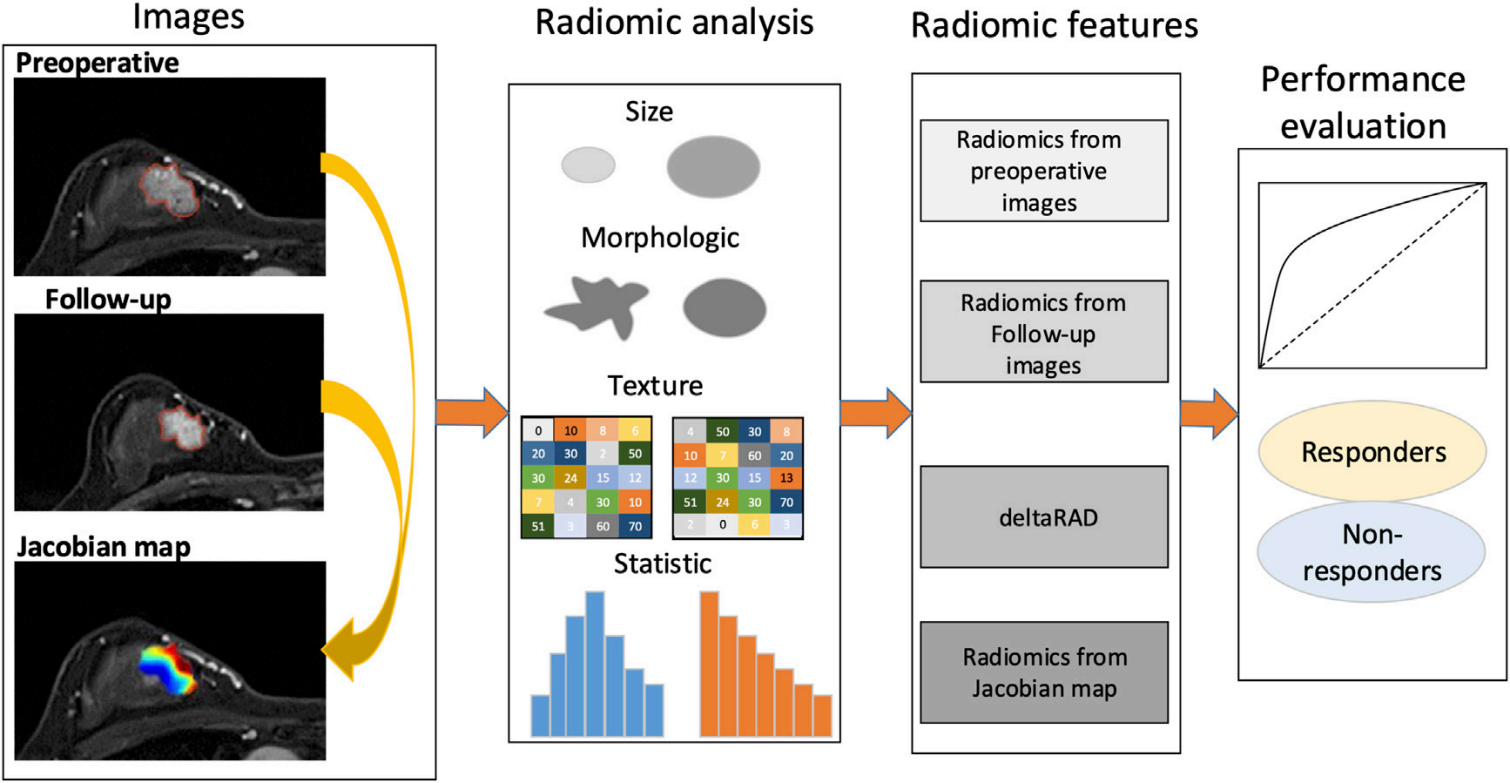
Study framework. A Jacobian map for each tumor was derived based on aligning the post-treatment images to the preoperative ones. Radiomics were calculated using the pre- and the follow-up images, the Jacobian map and the feature changes using longitudinal images (deltaRAD).

The framework of this study is illustrated in Figure 1. The voxelwise volumetric changes during treatment were evaluated to generate a Jacobian map by aligning the post-treatment MRI scans to the baseline ones. Radiomic analysis was performed on the pre-and post-NACT images and the Jacobian map. Feature-level changes in tumor heterogeneity were obtained by calculating the relative net change (the percent change) in features between baseline and post-NACT scans. Predictive models were established using radiomics based on the evaluation of these longitudinal images to discriminate tumors that responded to NACT from those that did not.

### Patient Selection

**FIGURE 2:**
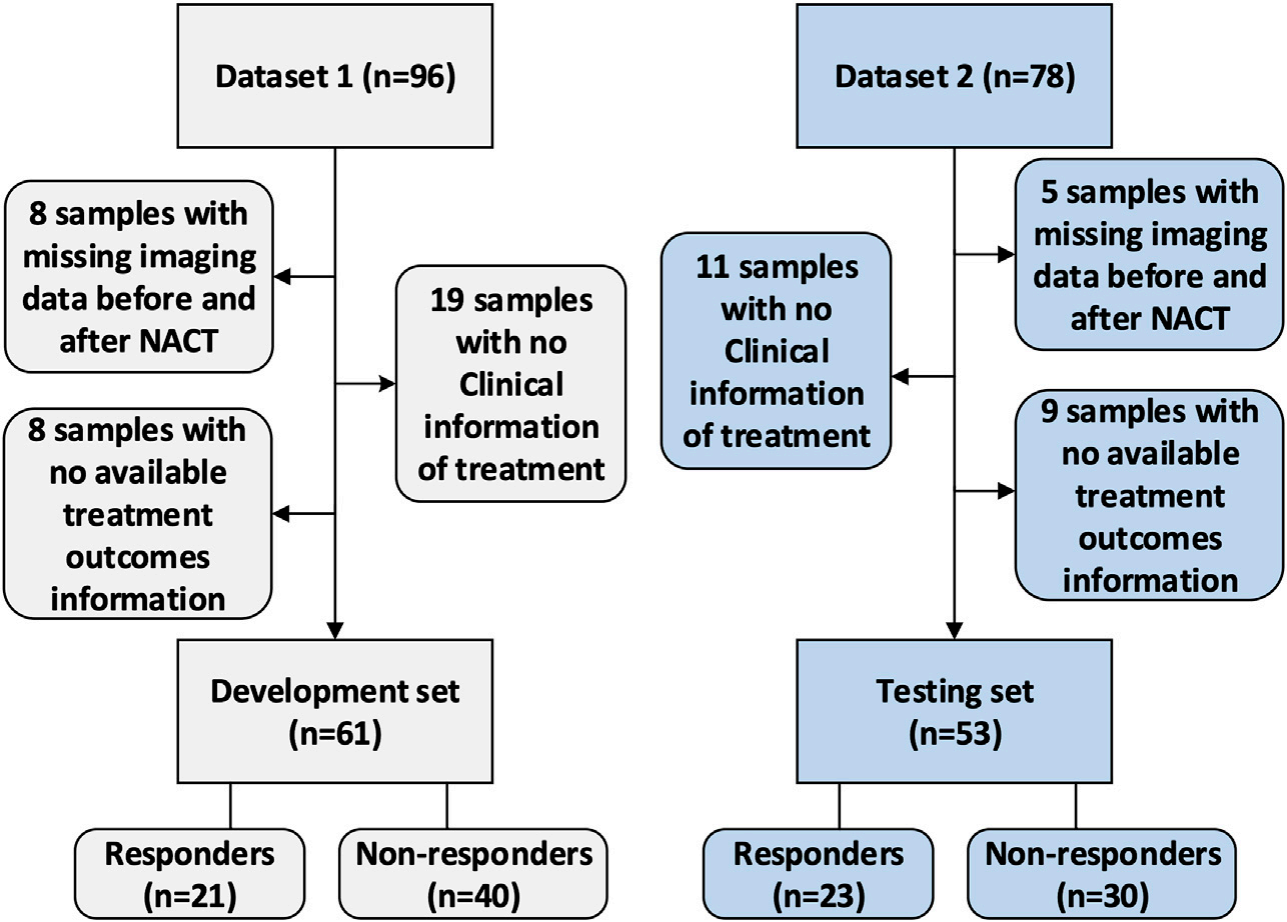
Data selection procedure.

This study was approved by the Institutional Review Board of Fudan University Shanghai Cancer Center. Due to the retrospective nature of this study, use of a consent form was waived. The data collection and selection procedure in these two cohorts are illustrated in Figure 2. The original dataset collected from the hospital included 174 samples with paired images acquired at the baseline and post-treatment (after two cycles of NACT). Dataset 1 (the development set) initially included 96 samples. After excluding eight samples with missing imaging sequences at baseline or after early NACT, eight samples with no available treatment outcome data evaluated by the Miler-Payne (MP) score, and 19 samples with no available clinical information, 61 samples were retained in this study. Dataset 2 (the testing set) initially included 78 samples, of which 25 were excluded: 11 with no clinical information, five with incomplete imaging sequences, and nine with no available MP data. The remaining data included 53 samples for testing.

### Data Analysis

Pathological response was assessed after the whole cycle of NACT according to the surgical specimen-determined MP grading system by comparing with the preoperative core biopsy (Ogston et al., 2003). This grading system includes five grades. According to a previous study, tumors with MP scores four and five (total cell loss of more than 90%), also termed almost pCR and pCR, respectively, were grouped as responders, while the others (grades 1, 2 or 3 with a total cell loss of up to 90%) were grouped as nonresponders (Zhu et al., 2014). Estrogen receptor (ER), progesterone receptor (PR) and Ki-67 status were defined according to immunohistochemistry (IHC) with streptavidin-peroxidase (SP) detection (Hammond et al., 2010; Wolff et al., 2013). Hormone receptor (HR) positivity was defined as HR and/or ER positive. HER2 positivity was defined as IHC score of 3+ or 2+ with confirmation of gene amplification by fluorescence *in situ* hybridization (FISH) (Wolff et al., 2013). Tumor subtypes were categorized as follows: luminal A (HR-positive and HER2-negative), luminal B (HR-positive and HER2-positive), HER2-enriched (HR-negative and HER2-positive) and triple-negative (HR-negative and HER2-negative) subtypes. The HR-positive and HER2-negative tumors with a Ki-67 expression level higher than 14% were specifically determined to be luminal B subtype tumors.

### Imaging Protocols

Imaging was performed following the specific requirements of the hospital. For the development dataset (*n* = 61), the images were acquired using a 3.0-T scanner (Siemens Healthcare, Erlangen, Germany). DCE-MRI was acquired with a fat-suppressed T1-weighted imaging sequence, which generated one precontrast (S0) followed by five or eight sequential postcontrast image series after injection of a gadobutrol-based contrast agent. The time interval between the first postcontrast image and S0 was 90 s, while the time intervals between the subsequent image series were 43 or 44 s.

For the testing dataset (*n* = 53), DCE-MRI was acquired using a dedicated 1.5-T breast magnetic resonance imaging system (Aurora Dedicated Breast MRI Systems, United States). The imaging system generated one precontrast image and three postcontrast images at 120, 245, and 371 s after beginning the intravenous administration of gadobutrol injection. The detailed imaging parameters for these two datasets are shown as in Table 1.

**TABLE 1:** Imaging parameters in the development and testing datasets.

Parameter	Development dataset	Testing dataset
Repetition time (TR) [ms]	4.5	29
Echo time (TE) [ms]	1.56	4.8
Flip angle (FA) [°]	10	90
Field of view (FOV) [mm]	360 × 360	360 × 360
Matrix	384 × 384	512 × 512
Slice thickness (mm)	2.2	1.48
In-plane resolution (mm)	0.9375 × 0.9375	0.7031 × 0.7031

### Image Preprocessing

Nonuniform intensity normalization (N4) bias correction was implemented to reduce the effect of MR imaging artifacts. Images from the patients were resampled to the same spatial resolution for feature extraction. The tumor region-of-interest (ROI) was identified by using a spatial Fuzzy C-means method on the third postcontrast image series where the highest enhancement valuate were usually achieved (Yang et al., 2014; Fan et al., 2020).

### Analysis of Volumetric Change in Longitudinal MRI Scans

Voxelwise volumetric changes were evaluated by aligning the follow-up images to preoperative scans by finding an optimal and deformable transformation for image registration (Ou et al., 2015). Based on this approach, the aligned image along with a Jacobian map was generated, in which each pixel of the Jacobian map represented a volumetric shrink/expansion pattern. Specifically, Jacobian values for each voxel greater than one indicate volume expansion, while those less than one indicate volume shrinkage, and those equal to one indicate volume preservation. The Jacobian value is calculated using the following equation (Eq. 1):

$$Jacobian\;value\;=\frac{v_{follow-up}}{v_{baseline}}$$ (1)

where $v_{2}$ denotes the resisted voxel volume in follow-up image, and $v_{1}$ denotes the voxel volume in the baseline image.

### Radiomic Features

Features were extracted from the tumor ROI using a publicly available radiomics analysis software, Pyradiomics (van Griethuysen et al., 2017). For each ROI, 102 features were calculated, including the shape (*n* = 14), first-order statistics (*n* = 18), texture features using gray level cooccurrence matrix (GLCM) (*n* = 24), gray-level run-length matrix (GLRLM) (*n* = 16), gray-level size-zone matrix (GLSZM) (*n* = 16) and gray-level dependence matrix (GLDM) (*n* = 14). The imaging heterogeneity of the entire tumor was evaluated based on the subtraction images between the intermediate image series that unusually exhibited the maximum enhancement signal and the precontrast image. Radiomics features were calculated on the pre- and post-treatment images and the Jacobian map.

### Feature-Level Changes in Tumor Heterogeneity

Feature-level changes were calculated by the relative net change between the features derived from the baseline and the follow-up image. For the *i* ^th^ radiomics feature ($f^{i}$) calculated from the tumor ROI, the feature change $f_{\Delta }^{i}$ is illustrated as shown in the following equation (Eq. 2):

$$f_{\Delta }^{i}\;=\frac{f_{baseline}^{i}-f_{follow-up}^{i}}{f_{baseline}^{i}}$$ (2)

where $f_{baseline}^{i}$ stands for the feature *i* obtained from the baseline image, and $f_{follow-up}^{i}$ indicates the feature *i* from the follow-up image.

### Statistical Analysis and Machine Learning Methods

The distributions of the histopathological information of the molecular subtypes, menopausal status, family history between the development and testing groups were compared by using the *χ* ^2^ test or Fisher’s exact test when the expected frequency in any tablet was less than five. Analysis of variance (ANOVA) was performed to compare continuous variables between the development and the testing groups. The area under the receiver operating characteristic (ROC) curve (AUC) was calculated to assess the performance of the predictive model. The sensitivity, specificity, positive predictive value (PPV) and negative predictive value (NPV) were calculated. The sensitivity and specificity were determined at the operation point at ROC curve by using the Youden index by the maximum sum of the specificity and the sensitivity. Statistical tests with *p* values less than 0.05 were considered significant.

A support vector machine (SVM) with a Gaussian kernel was used as a base classifier for prediction. Predictive model establishment and model tuning were performed on the development set and were tested on the testing set. SVM-recursive feature elimination (RFE) was used to rank the features that were most relevant to the target, and these were then sequentially added into the predictive model. The feature sets were fed into the predictive model, in which the SVM parameters *α* and *γ* were tuned using a grid search method in each iteration with a 10-fold cross-validation framework. An optimized model with the selected feature subset and the tuned model parameters was established using all the samples in the development set and was applied to the testing set to evaluate the model performance. Statistical analysis and machine learning methods were performed using R (version 4.0) and Matlab (MathWorks, Natick, Massachusetts, version 2018 b).

## Results

### Patient

Patient characteristics including age, menopausal status, family history, molecular subtypes and MP grade are illustrated in Table 2. The development dataset included 61 samples (mean age 49, ranges from 27 to 66°years), while the testing dataset included 53 samples (mean age 47, range from 29 to 79°years). There were 44 (38.6%) patients who had an MP grade larger than three (i.e., 4, 5), and they were categorized as the responders, while the others (*n* = 70, 61.4%) who had an MP grade of no more than three (i.e., 1, 2 or 3) were defined as the nonresponders. No significant differences in histological information were observed between the development and testing datasets (*p* > 0.05, Table 2).

**TABLE 2:** Patient characteristics.

	All	Development set	Testing set	*p*-value
Number	114	61 (54%)	53 (46%)	
Age	48 (27–79)	49 (27–66)	47 (29–79)	0.407^a^
Menopausal status				0.670^b^
Pre	46 (40%)	23 (38%)	23 (43%)	
Post	68 (60%)	38 (62%)	30 (57%)	
Family history				0.642^b^
No	87 (76%)	45 (74%)	42 (79%)	
Yes	27 (24%)	16 (26%)	11 (21%)	
Miller Payne				0.706^c^
1	9 (8%)	6 (10%)	3 (6%)	
2	21 (18%)	10 (16%)	11 (21%)	
3	40 (35%)	24 (40%)	16 (30%)	
4	10 (9%)	5 (8%)	5 (9%)	
5	34 (30%)	16 (26%)	18 (34%)	
Molecular subtypes				0.409^b^
Luminal A	12 (10%)	9 (15%)	3 (5%)	
Luminal B	58 (51%)	30 (49%)	28 (53%)	
Basal-like	20 (18%)	9 (15%)	11 (21%)	
HER-2	24 (21%)	13 (21%)	11 (21%)	

^a^ Analysis of variance.

^b^ *χ* ^2^ test with Yates’ continuity correction.

^c^ Fisher’s exact test.

### Voxelwise Changes in Tumor Heterogeneity Associated With the Response to NACT

**FIGURE 3:**
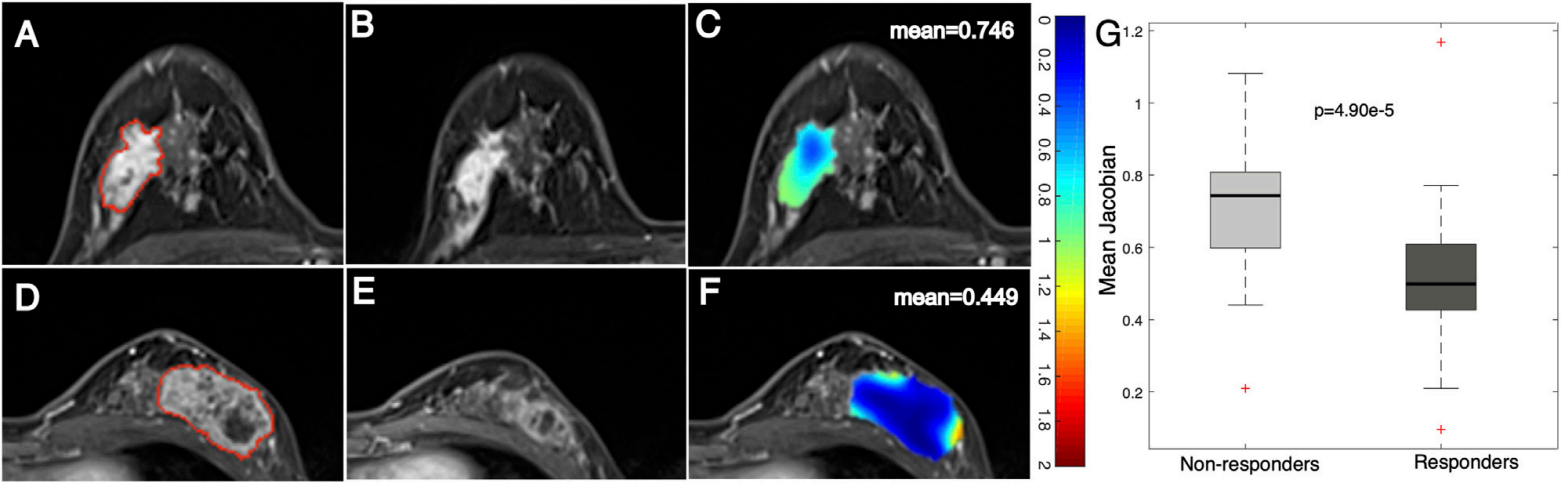
Example images and distribution of mean Jacobian values in nonresponders and responders. Images from a breast cancer patient (aged 45 years old) with a low MP (nonresponder) **(A)** pre- and **(B)** post-treatment and **(C)** a Jacobian map of the ROI (mean Jacobian value = 0.746). Images from a breast cancer patient (aged 41 years old) with a high MP (responder) **(D)** pre- and **(E)** post-treatment and **(F)** a Jacobian map of the ROI (mean Jacobian value = 0.449). **(G)** Boxplot representing the feature distribution between nonresponders and responders.

After registering the follow-up images to the baseline ones, a Jacobian map was obtained for each tumor that reflects the level of voxelwise volumetric shrink/expansion. An example of a statistical feature (e.g., mean value) calculated on the Jacobian map of tumors is illustrated in Figure 3. Tumor volume was reduced in both the nonresponse (Figures 3A−C) and the response (Figures 3D−F) groups after NACT. The mean Jacobian value inside the tumor was significantly higher in the nonresponders than in the responders, with a *p* value of 4.9e^−5^ (Figure 3G). This result indicated that a high Jacobian value that represents a lower level of voxelwise shrink inside a tumor is associated with a failure of treatment.

**FIGURE 4:**
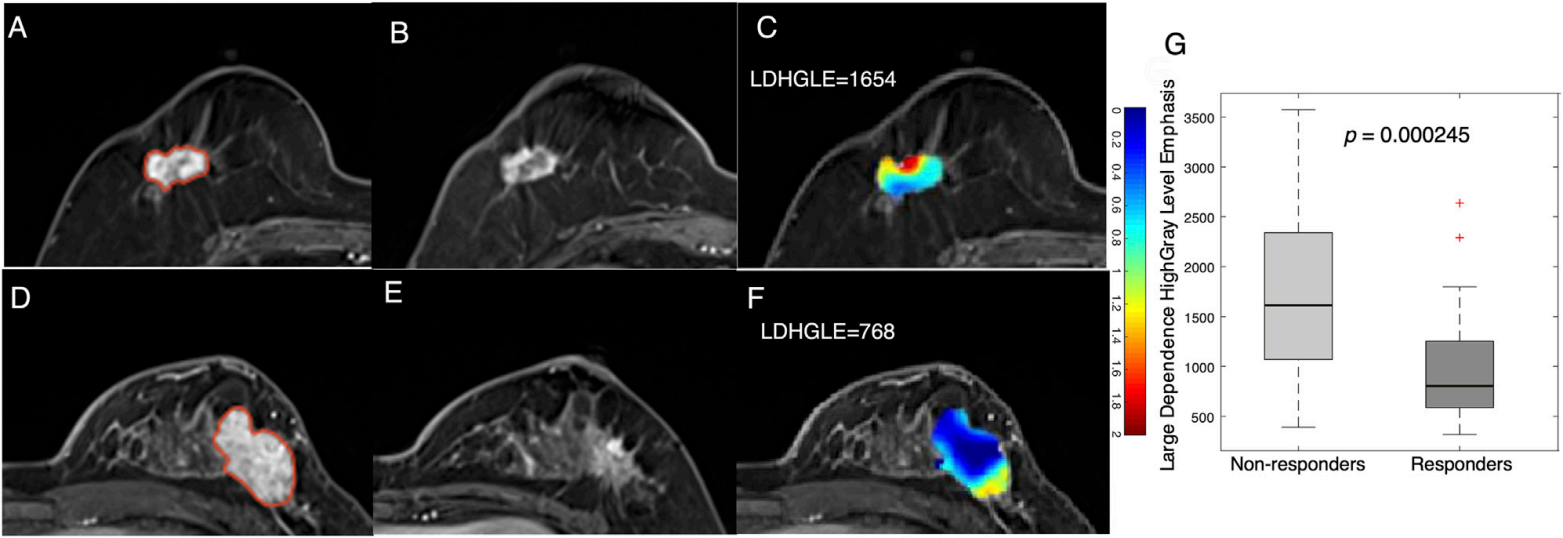
Examples feature of large dependence high gray-level emphasis (LDHGLE) in nonresponders and responders. Images from a nonresponder breast cancer patient (aged 59 years old) **(A)** pre- and **(B)** post-treatment and **(C)** a Jacobian map of the tumor ROI (LDHGLE = 1654). Images from a responder breast cancer patient [aged 43 years old) **(D)**] pre- and **(E)** post-treatment and **(F)** a Jacobian map of the tumor ROI (LDHGLE = 768). **(G)** Boxplot representing the feature distributions in nonresponders and responders.

In addition to statistical features, examples of texture features derived from tumor Jacobian maps are illustrated in Figure 4. A low MP grade (nonresponder) patient showed a lower level of volume shrinkage (Figures 4A,B) after early NACT than a patient with a higher MP grade (responder) (Figures 4D,E); this pattern is illustrated in the Jacobian map (Figures 4C,F). The texture feature (large dependence high gray-level emphasis) obtained from the Jacobian map were significantly higher in the nonresponders than in the responders (Figure 4G, *p* = 2.45e^−4^). This result suggested that a higher level of this texture feature, which reflects a higher voxelwise spatial rearrangement heterogeneity of the shrinkage/expansion pattern inside a tumor during NACT, is more likely to be associated with a worse response to NACT.

### Feature-Level Changes in Tumor Heterogeneity Associated With Response to NACT

To assess how the tumor heterogeneity changed during treatment, radiomics analysis was conducted on the baseline and the follow-up images. It should be noted that the relative net change in volume size for the entire tumor between pre- and post-treatment images showed no significant (*p* = 0.09) differences between the responders and nonresponders (Supplementary Figure S1). This result suggests that volumetric changes in the entire tumor after early NACT may not be significantly related to the eventual treatment outcomes.

**FIGURE 5:**
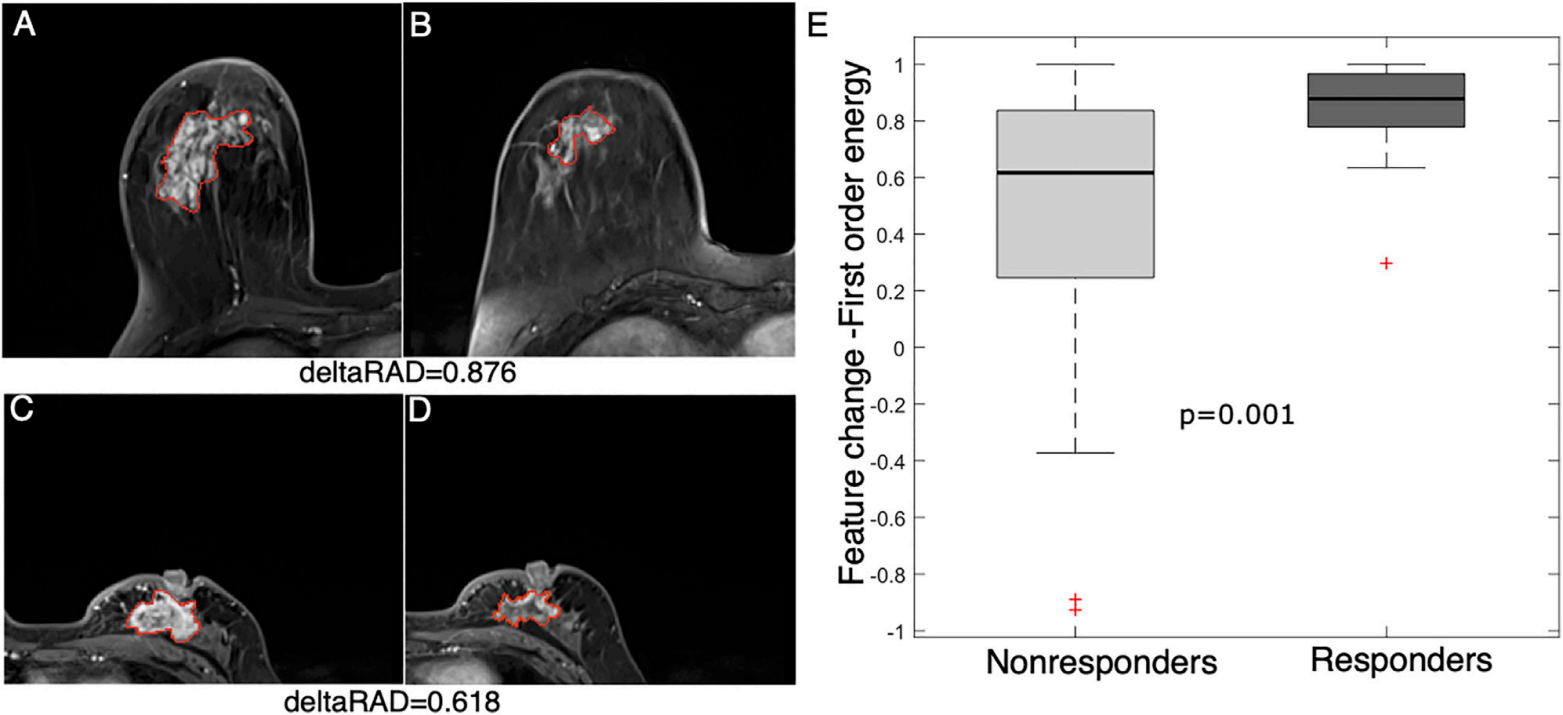
Feature (energy) change between baseline and early NACT images. Images from a 58 year-old woman with a deltaRAD value of 0.876 in the responders at **(A)** baseline and **(B)** early NACT. Images from a 49 year-old woman with a deltaRAD value of 0.618 in the nonresponders at **(C)** baseline and **(D)** early NACT. **(E)** The distribution of the change in the energy value is shown in the boxplot, in which the feature value is significantly higher in responders than in nonresponders.

A more significant (*p* = 0.001) decrease in a statistical feature (energy) after early NACT was observed in the responders than in the nonresponders (Figure 5). This feature measures the magnitude of voxel values, and a higher value suggests a greater sum of the squares of these values. The result suggests that a decrease in the enhancement level of tumors is associated with NACT response in breast cancer.

**FIGURE 6:**
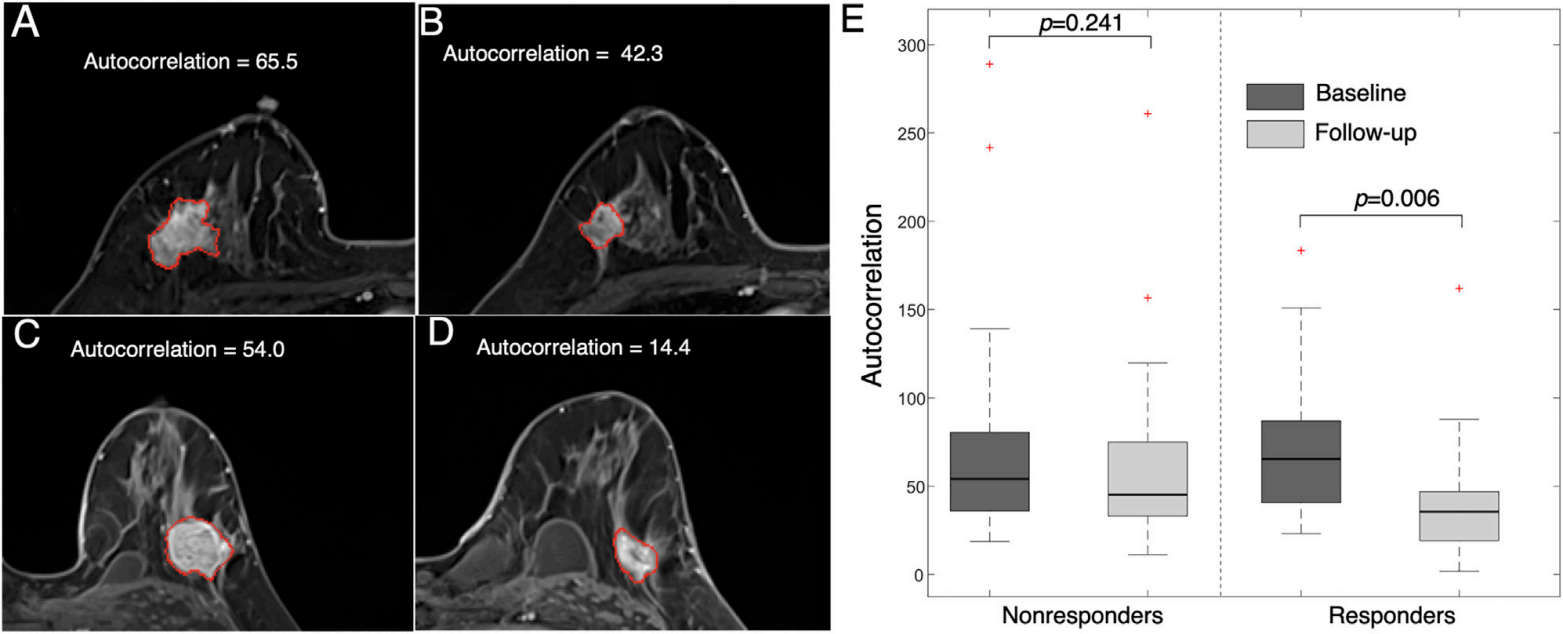
Images representing feature (autocorrelation) changes between pre- and post-treatment images. Images from a 47 year-old woman who responded to NACT (high MP grade) at **(A)** baseline (autocorrelation = 65.5) and **(B)** follow-up (autocorrelation = 42.3). Images from a 36 year-old woman who did not respond to NACT (low MP grade) at **(C)** baseline (autocorrelation = 54.0) and **(D)** follow-up (autocorrelation = 14.4). **(E)** Boxplot showing that the feature value is significantly reduced in responders (*p* = 0.006) but is not significantly changed in nonresponders (*p* = 0.241).

An example of a texture feature (i.e., autocorrelation) is also illustrated in Figure 6. This feature value was significantly reduced after early NACT in the responders (*p* = 0.006), while the difference was not significant in the nonresponders (*p* = 0.241). This feature measures the level of the fineness and coarseness of the texture of an object, in which a high value is correlated with high gray-level heterogeneity within the tumor. The results suggest that the level of tumor heterogeneity reduction is higher in the NACT responders than in the nonresponders.

### Fusion of Longitudinal MRI Features for Predicting Response to NACT

To evaluate the collective effect of longitudinal radiomics, the features from different images were combined and evaluated. The individual features from the images at baseline, post-treatment, Jacobian map and deltaRAD features were evaluated, and the results showed that features from the follow-up image have the highest performance (in terms of AUC values), while the deltaRAD features and Jacobian map-based features showed intermediate performance (Supplementary Figure S2).

**FIGURE 7:**
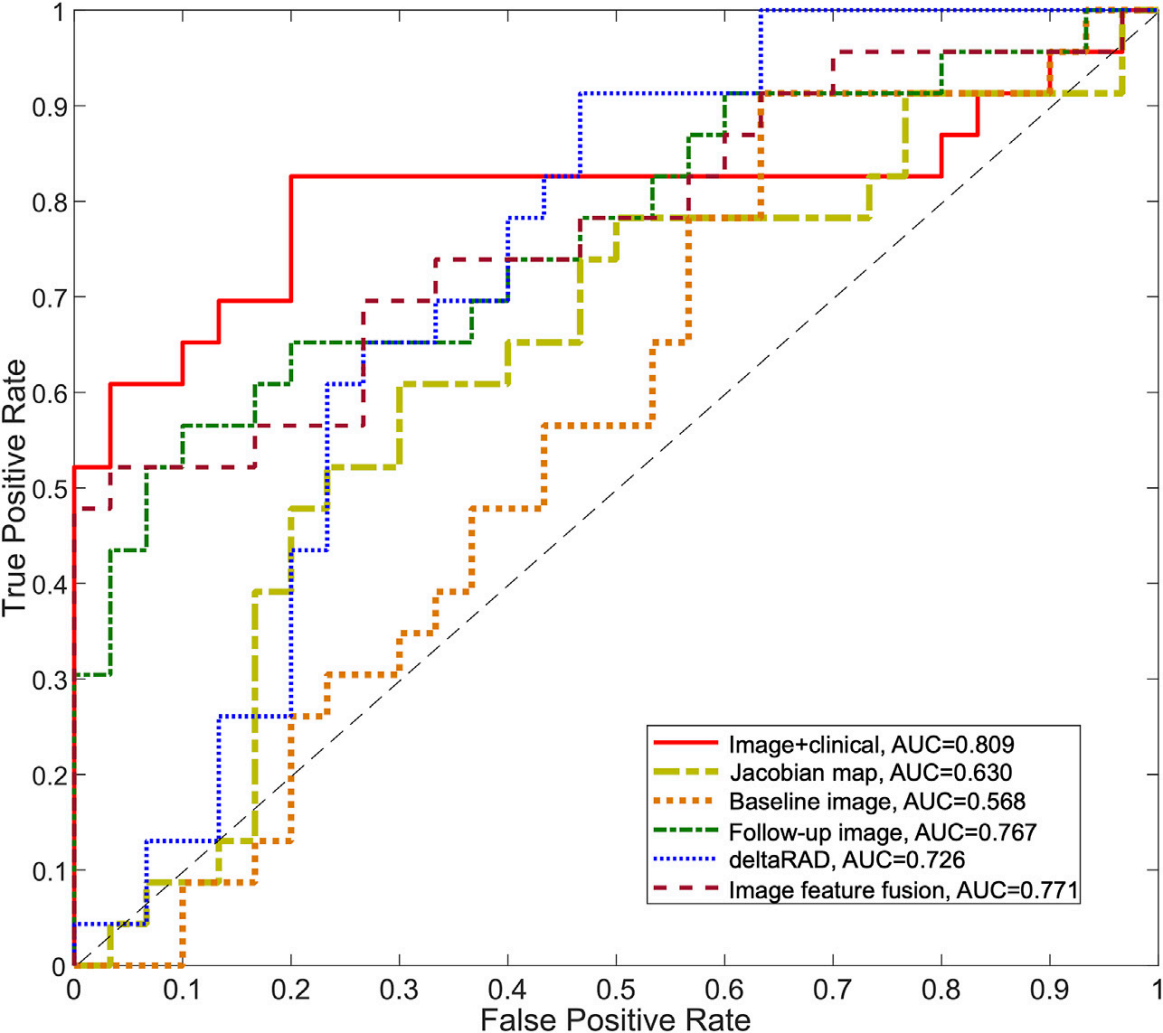
ROC curves for the predictive models using longitudinal images. The ROC curves for the predictive model using deltaRAD and radiomics derived from pre- and post-treatment images, the Jacobian map and the fused imaging features are shown. The ROC curve of the predictive model combining imaging features and molecular subtype information is also shown.

Radiomic features from these images were used separately to establish predictive models in the development set and was tested on the testing set (Table 3; Figure 7). Among these, the predictive model based on the baseline image generated lowest performance with an AUC of 0.568 (sensitivity of 0.913 at a specificity of 0.367). Radiomic features based on Jacobian map, follow-up image and deltaRAD showed a higher prediction performance with AUC of 0.628, 0.757 and 0.718, respectively. When the features from these images were fused, the classifier generated an AUC of 0.771 with sensitivity of 0.522 and specificity of 0.967. Finally, imaging features were combined with the clinical and histologic information for prediction to facilitate a more accurate prediction. The results showed an improved performance with an AUC value of 0.809 (sensitivity of 0.826 at a specificity of 0.800), which is significantly better than the baseline image (*p* = 0.028) and the Jacobian map (*p* = 0.019) based predictive model.

**TABLE 3:** Performance of predictive model based on images at longitudinal times.

Images	AUC (±SE)	SD	*p* value	Sensitivity	Specificity	PPV	NPV
Baseline image	0.568 ± 0.155	0.079	0.028	0.913	0.367	0.525	0.846
Follow-up image	0.767 ± 0.128	0.065	0.508	0.565	0.900	0.813	0.730
DeltaRAD	0.726 ± 0.137	0.070	0.301	0.913	0.533	0.600	0.889
Jacobian map	0.630 ± 0.154	0.079	0.019	0.609	0.700	0.609	0.700
Feature fusion	0.771 ± 0.136	0.069	0.356	0.522	0.967	0.923	0.725
Feature + MS	0.809 ± 0.131	0.067	—	0.826	0.800	0.760	0.857

SE, standard error; SD, standard derivation; deltaRAD, relative net feature change between baseline and follow-up images; MS, molecular subtype. *p* value indicates significance of the comparison between baseline imaging- and the other image-based predictive models.

## Discussion

During NACT, the breast tumor size and morphological and functional changes are associated with the eventual treatment outcomes. In this study, the pattern of the changes in tumor heterogeneity during NACT was evaluated using baseline and post-treatment images to predict responses to NACT in breast cancer. The voxelwise shrinkage/expansion inside the tumor and the feature-level changes of the entire tumor were both obtained. Radiomics features from longitudinal images and the changes in tumor heterogeneity were fused for the prediction. The molecular subtype information was combined with radiomics features, which generated an increased prediction performance.

Previous studies have conducted radiomic analysis using features derived from tumors for NACT response prediction. Jahani et al. analyzed voxelwise changes in DCE-MRI features to characterize heterogeneous changes within the tumor and to predict pCR and recurrence free survivals (Jahani et al., 2019). An earlier study evaluated image feature-level changes in tumor heterogeneity to assess for pCR to NACT (Parikh et al., 2014). In our study, radiomic features based on changes in tumor heterogeneity were evaluated in both feature- and voxel-level to facilitate a quantitative analysis of longitudinal heterogeneity during treatment in breast cancer. A recent study extracted texture and statistical features and identified that tumor kurtosis in T_2_-weighted MR images was independently associated with pCR in non-triple-negative breast cancer (Chamming’s et al., 2018). Additionally, the molecular subtypes were associated with the prediction accuracy of NACT response (Drisis et al., 2016; Liu et al., 2019). In this study, we have incorporated molecular subtype information in the predictive model and observed the highest performance, which is partly consistent with previous study.

In this study, radiomics analysis of Jacobian maps showed that statistical features (e.g., mean) and texture features (e.g., large dependence high gray level emphasis) decreased after early NACT, while the level of feature reduction was lower in the responders than in the nonresponders. On the other hand, the voxelwise volumetric reduction inside tumors was significantly associated with the responders. Additionally, texture features (e.g., large dependence high gray-level emphasis) were reduced after early NACT, and the level of the reduction was higher in patients who responded to NACT than in those who did not. In our results, tumor heterogeneity was decreased after early NACT, and more importantly, the high level of reduction in heterogeneity was associated with good response to NACT. This indicated that decreased heterogeneity within a tumor may likely be exhibit in the patients who benefitted from the NACT.

In addition to the evaluation of voxel-vize volumetric changes by image registration, longitudinal feature-level changes between the baseline and follow-up images were also evaluated for their associations with tumor response to NACT. In our study, the performance of the model based on vascular characteristics measured by DCE-MRI was higher than that of the model based on morphologic features, which is partly consistent with the findings of a previous study that dynamic features have better accuracy in response prediction than tumor size (Marinovich et al., 2012). Our results indicated that tumor heterogeneity-related features are decreased after treatment, and the extent is higher in responders than in nonresponders. Therefore, longitudinal feature changes in tumor heterogeneity, rather than size changes of the entire tumor, might be more correlated with tumor response to NACT.

We observed a relatively lower performance in terms of AUC for features from the baseline images. A related study identified significant change in the tumor maximum diameter between the responders and nonresponders (Minarikova et al., 2017). In our study, changes in tumor heterogeneity at the feature level and voxel level were both evaluated, and predictive performance was improved after fusing the features from different images at varied times. The results suggested that multiple levels of features and different stages of features at treatment may be complementary, and altogether, these contributed to enhanced model performance.

Despite the potential significance of tumor radiomics using longitudinal images in this study, several limitations should also be addressed. First, only the tumor region was analyzed for image feature extraction. It would also be interesting to analyze the peritumoral tissues that surrounds the tumor (Kim et al., 2018b) to conduct a comprehensively analysis of the pattern of heterogeneity on baseline and post-treatment images. Second, this was a retrospective study, and the sample size was relatively small to conduct a fair statistical analysis. Further studies with more samples and refined analyses should be conducted to confirm the findings of this study. Third, features were derived from two datasets with different magnetic field strengths (3.0 and 1.5 T for the development and testing datasets, respectively), which may have affected the feature calculations and induced bias. Despite this limitation, the features were calculated based on the relative differences in the feature/voxel values between baseline and follow-up images, which may have partly reduced the bias between different protocols. In our study, radiomics features were calculated using publicly available Pyradiomics software, with the aim of ensuring the repeatability of this study (van Griethuysen et al., 2017).

In conclusion, longitudinal changes in tumor heterogeneity at the voxel and feature levels were examined to determine their contribution to the prediction of tumor response. It was found that molecular subtypes add more predictive power in assessing the response to NACT.

## Data Availability

The original contributions presented in the study are included in the article/Supplementary Material, further inquiries can be directed to the corresponding author.
