# Supplementary material for: Radiomics of Tumor Heterogeneity in Longitudinal Dynamic Contrast-Enhanced Magnetic Resonance Imaging for Predicting Response to Neoadjuvant Chemotherapy in Breast Cancer
Source: Front Mol Biosci. 2021 Mar 22;8:622219. doi: 10.3389/fmolb.2021.622219 (PMC8044916; doi:10.3389/fmolb.2021.622219)
Supplement: Supplementary file 1 [file datasheet1.docx]

Supplementary Figure 1. Distribution of tumor volume in the nonresponders and responders

Supplementary Figure 2. Individual feature performances in terms of AUCs
